# Psychological effects of a disastrous hydrogen fluoride spillage on the local community

**DOI:** 10.1186/s40557-017-0196-6

**Published:** 2017-09-11

**Authors:** Seung-Hyun Yoo, Seong-Yong Yoon, Kuck-Hyun Woo, Jin-Seok Kim, Seong-Yong Cho, Sung-Soo Lee, Hyun-Sul Lim, Yeon-Soon Ahn, Won-Ho Yang

**Affiliations:** 1 Department of Occupational and Environmental Medicine, Soonchunhyang University Gumi Hospital, 179, Gongdan 1-dong, Gumi-si, Gyeongbuk 730-706 South Korea; 2 Department of Preventive Medicine, Soonchunhyang University School of Medicine, 23-20, Bongmyeong-dong, Cheonan-si, Chungcheongnam-do 330-721 South Korea; 3 Department of Preventive Medicine, Dongguk University College of Medicine, 87 Dongdae-ro, Seokjang-dong, Gyeongju-si, Gyeongbuk 780-350 South Korea; 4 Department of Occupational and Environmental Medicine, Dongguk University Ilsan Medical Center, 27, Dongguk-ro, Ilsandong-gu, Goyang-si, Gyeonggi-do South Korea; 5 Department of Occupational Health, Catholic University of Daegu, Hayang-Ro 13-13, Hayang-Eup, Gyeongsan-si, Gyeongbuk South Korea

**Keywords:** Hydrogen fluoride, Disaster, Psychological effect

## Abstract

**Background:**

On September 27, 2012, at 3:43 pm, a hydrogen fluoride spill occurred in a manufacturing plant located at the 4th complex of the Gumi National Industrial Complex in Gumi City, South Korea. The present study aimed to evaluate the psychological effects of the hydrogen fluoride spill on the members of the community and to investigate their relationships with physical symptoms and changes in psychological effects occurring as time passed after the accident.

**Methods:**

The 1st phase involved a survey of 1359 individuals that was conducted 1 month after the spill, and the 2nd phase involved a survey of 711 individuals that was conducted 7 months after the accident. The questionnaires included items for assessing demographic characteristics, hydrogen fluoride exposure level, physical symptoms, and psychological status. Physical symptoms were assessed to determine the persistence of irritations. Psychological status was assessed to investigate the impact of event level using the Impact of Event Scale – Revised Korean version (IES-R-K), and the anxiety level was assessed using the Beck Anxiety Inventory (BAI).

**Results:**

As the hydrogen fluoride exposure level increased, the impact of event and anxiety levels increased significantly both 1 and 7 months after the accident (*p* < 0.05). The mean score of the impact of event levels decreased significantly from 33.33 ± 14.64 at 1 month after the accident to 28.68 ± 11.80 at 7 months after the accident (*p* < 0.05). The mean score of the anxiety levels increased significantly from 5.16 ± 6.59 at 1 month after the accident to 6.79 ± 8.41 at 7 months after the accident (*p* < 0.05). The risk of persistent physical symptoms at 7 months after the accident was significantly higher in females. The risk of persistent physical symptoms also increased significantly, with increasing age, hydrogen fluoride exposure, and impact of event levels (*p* < 0.05).

**Conclusions:**

The present study found that the impact of event level and anxiety level increased with increasing hydrogen fluoride exposure. Anxiety levels persisted even after time passed. The risk of persistent physical symptoms at 7 months after the accident was higher in females, and it increased with increasing age, hydrogen fluoride exposure level, and impact of event levels.

## Background

On September 27, 2012, at 3:43 pm, a spill occurred at a hydrogen fluoride manufacturing plant located in the 4th complex of the Gumi National Industrial Complex while a 100% hydrogen fluoride solution was being transferred from a tank truck to a storage tank inside the plant. An estimated 8—12 tons of hydrogen fluoride was leaked for about 8 h until the tank spill was completely stopped. The accident killed 5 workers either onsite or during transport, and hydrogen fluoride spread through the air into the communities, causing physical and psychological damage to the local residents in nearby villages and workers in the industrial complex [1].

Hydrogen fluoride combines with moisture in the mucous membranes of the human body, causing irritation first in the skin, eyes, and the upper respiratory system, including the nose and throat. When a large amount of hydrogen fluoride gas is inhaled, it may penetrate into the lower respiratory tract as well, causing interstitial pneumonia and pulmonary edema. In addition, when the gas is absorbed by the gastrointestinal tract, it can cause gastrointestinal symptoms such as nausea and vomiting [2–4]. In a study analyzing 1890 outpatients and 12 hospitalized patients at a hospital near the Gumi hydrogen fluoride spill site, the chief complaints of the outpatients included sore throat, headache, cough, and eye irritation, all symptoms caused by hydrogen fluoride gas. Of a total of 12 hospitalized patients, 11 were discharged within 1 week, and the chief complaints of the hospitalized patients included respiratory symptoms such as hemoptysis and dyspnea, gastrointestinal symptoms such as nausea and indigestion, neurologic symptoms such as headache and numbness, and other symptoms including sore throat and lip pain. Patients who were closer to the accident site had shortness of breath and sputum more often than those who were further away from the accident site [5].

The category of disasters includes natural disasters such as floods, typhoons and earthquakes, large-scale pollution accidents such as oil spills and chemical spills, and large-scale traffic accidents. Although the degree of physical damage differs depending on the nature of the disaster, all disasters have common psychological effects on the disaster victims. Disaster victims may present with persistent anxiety and depression in response to severe stress, and they may develop post-traumatic stress disorder (PTSD). Disaster victims may also present with non-specific symptoms such as headache, dyspepsia, and dizziness associated with such psychological problems [6].

There have been many studies on the psychological effects of disasters on the victims. A study of the 1980 Mount St. Helens volcanic eruption victims found that the incidences of PTSD, generalized anxiety disorder, and major depression increased in both male and females in accordance with the extent of risk exposure [7]. A study on the psychological effects of the Exxon Valdez oil spill in 1982 on the local residents investigated the relationship between the incidence rates of PTSD and generalized anxiety disorder and the extent of oil exposure. The results of that study found that the high exposure group was at a 2.8-fold higher risk of developing PTSD and a 3.6-fold higher risk of developing generalized anxiety disorder one year after the accident, compared to the non-exposure group [8]. In a South Korean study of the health effects of the 2007 Hebei Spirit oil spill accident on the local residents, the related psychological effects were assessed via anxiety and depression level measurements. The percentage of residents with severe anxiety or depression in highly polluted areas was found to be about 2-fold higher than that in areas with low pollution [9].

The disaster resulting from the hydrogen fluoride spill caused physical and psychological damage to local residents in nearby areas as well as industrial workers. In addition, the widespread damage to crops, plants, and facilities increased the community’s anxiety. However, since the hydrogen fluoride spill was first experienced in the country, a psychological approach to the accident was insufficient for relieving the anxiety of local residents during the early stages of the accident. Therefore, the present study aimed to evaluate the psychological effects of the hydrogen fluoride spill disaster on local residents within the community and examine its relationship with physical symptoms and changes with the passage of time, with reference to previous studies.

## Methods

Health effect surveys were conducted in two phases after the hydrogen fluoride spill on September 27, 2012. The 1st phase survey was administered to a total of 1359 individuals, including 220 local residents living within a 1.5 km radius of the accident site, 829 local workers within a 1.5 km radius, and 310 Okgye East Middle School students and teachers within a 1.5–3 km radius from October 13, 2012 to January 12, 2013. The 2nd phase survey was conducted from February 19, 2013 to May 14, 2013 with 711 respondents (83 local residents, 413 workers in nearby area, and 214 Okgye East Middle School students and teachers) who completed a questionnaire that was mailed to 1359 subjects involved in the 1st phase survey.

The 1st phase health effect survey was conducted in a face-to-face manner, in which experienced interviewers visited each subject’s residence or workplace and called out questions and examples one by one for the subject to answer. The questionnaire consisted of items for assessing demographic characteristics, physical symptoms and psychological status, and items for assessing hydrogen fluoride exposure. Surveyed demographic characteristics were gender, age, and residence or workplace. The surveyed physical symptoms included the presence or absence of persistent irritation in the eyes, throat, and respiratory system one month after the accident. The Impact of Event Scale-Revised Korean version (IES-R-K), a psychological status assessment instrument, was used to assess the impact of event level, and the Beck Anxiety inventory (BAI) was used to assess anxiety level. The IES-R-K consisted of a total of 22 items, with 6 items on hyperarousal, 6 items on avoidance, 5 items on invasion, and 5 items on sleep disturbance, emotional paralysis, and dissociation symptoms. A score for each item ranged from 0 points for “not at all” to 4 points for “extremely,” depending on how an individual subject felt about each item, and the maximum total score was 88 points. With regard to assessment criteria for the impact of event level, individuals with a score of ≤24 points, those with a score of 25—39 points, those with a score of 40—59 points, and those with a score of ≥60 points were classified into the following groups: normal, moderate, severe, and very severe, respectively [10]. The BAI consists of 21 items including cognitive, emotional, and physical domains. Each item is scored from 0 points for “did not feel it at all” to 3 points for “felt it severely,” based on the participants’ responses, with a maximum total score of 63 points. With respect to the assessment criteria for anxiety level, subjects with a score of ≤21 points, those with a score of 22—26 points, those with a score of 27—31 points, and those with a score of ≥32 points were classified into the following groups: normal, anxiety, severe anxiety, and extreme anxiety, respectively [11].

Hydrogen fluoride exposure was assessed through related questionnaire items because there were no toxicity-related data available that measured individuals’ exposure at the time of the spill. Questions relating to evacuation (whether evacuated or not, post-accident evacuation time), return-related questions (whether returned or not, post-accident return time), subjective exposure level (respiratory exposure, subjective exposure for 24 h after the accident), questions related to the place of stay (indoor/outdoor, length of staying time), and environmental events (whether or not they witnessed the environmental corrosion at the time of the accident) were all used to assess the level of exposure. Scores were assigned based on each subject’s responses, and a total score was calculated for each subject to be classified based on the extent of hydrogen fluoride exposure. The maximum total score was 26 points. Exposure ratings were classified into four grades, the lowest being 0–25%, which was classified as extremely low exposure, 25–50% as low exposure, 50–75% as medium exposure, and 75–100% as high exposure [12].

The 2nd phase survey was conducted by mailing a self-administered questionnaire to all the subjects of 1st phase survey. Items concerning physical symptoms were used to determine whether eye, nose, and respiratory symptoms persisted up to 7 months after the accident. The IES-R-K and BAI scales were used as follow-up psychological status assessments.

All statistical analyses were performed using SPSS version 14.0 (SPSS, Inc., Chicago, IL, USA). The chi square test was performed to analyze the demographic characteristics of the subjects, the exposure level distribution and the proportion of subjects having persistent symptoms. An ANOVA was performed to compare IES-R-K and BAI scores according to hydrogen fluoride exposure level. McNemar’s test was performed to analyze changes in the distribution of the impact of event and anxiety levels 1 month and 7 months after the accident. A paired t-test was performed to compare the mean IES-R-K and BAI scores for all subjects 1 month and 7 months after the accident. Multiple logistic regression analysis was performed to determine the factors affecting the persistence of physical symptoms 7 months after the accident. The persistence or non-persistence of physical symptoms 7 months after the accident was used as a dependent variable. If one or more of the three symptoms persisted, these were identified as persistent physical symptoms. Age, gender, hydrogen fluoride exposure level, the impact of event level 7 months after the accident, and anxiety level 7 months after the accident were used as the independent variables.

## Results

The subjects’ demographic characteristics and the distribution of exposure levels are shown in Table 1. Concerning age group, the number of subjects under the age of 20 was 304 (22.4%) at 1 month after the accident and 202 (28.4%) at 7 months after the accident, and the number of those in their 30s was 306 (22.5%) at 1 month after the accident and 154 (21.7%) at 7 months after the accident, accounting for a high proportion of responses. The proportion of subjects in their 50s, 60s, 70s, and 80s was low (10% or less for each age group) at 1 month and 7 months after the accident. By gender, the proportion of male subjects at 1 month and 7 months after the accident was 836 (61.5%) and 444 (62.4%), respectively, and this was higher than the proportion of the female subjects. Concerning population group, the proportion of workers in nearby areas was the highest, with 829 (61.0%) and 413 (58.1%) at 1 month and 7 months after the accident. Concerning the distribution of the exposure level, the proportion of the extremely low exposure group was the lowest, with 90 (6.6%) and 52 (7.3%) at 1 month and 7 months after the accident, whereas the proportion of the low exposure group was the highest, with 463 (34.1%) and 237 (33.3%) at 1 month and 7 months after the accident (Table 1).

**Table 1:** Study subjects’ general characteristics

	1st survey (1 month)		2nd survey (7 months)		*P*-value^a^
Variable	*n*	(%)	*n*	(%)	
Age					0.092
< 20	304	(22.4)	202	(28.4)	
20–29	203	(14.9)	108	(15.2)	
30–39	306	(22.5)	154	(21.7)	
40–49	260	(19.1)	126	(17.7)	
50–59	136	(10.0)	59	(8.3)	
60–69	56	(4.1)	24	(3.4)	
70–79	70	(5.2)	31	(4.4)	
≥ 80	24	(1.8)	7	(1.0)	
Gender					0.679
Male	836	(61.5)	444	(62.4)	
Female	523	(38.5)	267	(37.6)	
Population					0.000
Middle school^b^	310	(22.8)	215	(30.2)	
Resident^c^	220	(16.2)	83	(11.7)	
Worker^d^	829	(61.0)	413	(58.1)	
Exposure level					0.920
Extremely low	90	(6.6)	52	(7.3)	
Low	463	(34.1)	237	(33.3)	
Moderate	430	(31.6)	229	(32.2)	
High	376	(27.7)	193	(27.1)	
Total	1359	(100.0)	711	(100.0)	

^a^p value for chi-square test

^b^workers and students of Okgye East Middle School

^c^residents living near the accident site

^d^workers employed by companies near the accident site

We compared the impact of event levels and anxiety levels according to hydrogen fluoride exposure at 1 month and 7 months after the accident. The mean IES-R-K score or the mean score of impact of event levels according to exposure level at 1 month after the accident was 26.06 ± 8.31 points, 30.32 ± 11.84 points, 34.82 ± 15.46 points, and 42.95 ± 18.09 points in the extremely low, low, moderate, and high exposure groups, respectively. The mean IES-R-K score according to exposure level at 7 months after the accident was 24.37 ± 4.32 points, 24.95 ± 6.75 points, 28.35 ± 12.37 points, and 34.51 ± 14.10 points in the extremely low, low, moderate, and high exposure groups, respectively. The mean IES-R-K score at 1 month and 7 months after the accident significantly increased in accordance with exposure level for everyone from the extremely low exposure group to the high exposure group. (*p* < 0.05) The mean BAI score or the mean score of anxiety levels by exposure level at 1 month after the accident was 3.66 ± 4.64 points, 5.00 ± 5.78 points, 5.21 ± 7.18 points, and 6.84 ± 8.21 points in the extremely low, low, moderate, and high exposure groups, respectively. The mean BAI score by exposure level 7 months after the accident was 4.24 ± 5.80 points, 4.16 ± 5.80 points, 6.77 ± 8.20 points, and 10.48 ± 9.84 points in the extremely low, low, moderate, and high exposure groups, respectively. The mean total BAI score 1 month after the accident significantly increased in accordance with exposure level for everyone from the extremely low exposure group to the high exposure group. (*p* < 0.05) The mean total BAI score 7 months after the accident significantly increased in accordance with exposure level for everyone from the low exposure group to the high exposure group. (*p* < 0.05) (Table 2).

**Table 2:** Comparison of mean IES-R-K and BIA scores after 1 month and 7 months

	Exposure level				
	Extremely low	Low	Moderate	High	*p*-value^a^
	Mean	Mean	Mean	Mean	
IES-R-K score 1 month (*n* = 1359)	26.06 ± 8.31	30.32 ± 11.84	34.82 ± 15.46	42.95 ± 18.09	0.000
IES-R-K score 7 month (*n* = 711)	24.37 ± 4.32	24.95 ± 6.75	28.35 ± 12.37	34.51 ± 14.10	0.000
BAI score 1 month (*n* = 1359)	3.66 ± 4.64	5.00 ± 5.78	5.21 ± 7.18	6.84 ± 8.21	0.000
BAI score 7 month (*n* = 711)	4.24 ± 5.80	4.16 ± 5.80	6.77 ± 8.20	10.48 ± 9.84	0.000
	Total subjects				
	1 month (*n* = 711)		7 month (*n* = 711)		*p*-value^b^
	Mean		Mean		
IES-R-K score	33.33 ± 14.64		28.68 ± 11.80		0.000
BAI score	5.16 ± 6.59		6.79 ± 8.41		0.000

^a^ *p* value for ANOVA test

^b^ *p* value for paired t-test

We compared changes in the impact of event levels and anxiety levels at 1 month and 7 months after the accident. The mean total IES-R-K score significantly decreased from 33.33 ± 14.64 points 1 month after the accident to 28.68 ± 11.80 points 7 months after the accident. (*p* < 0.05) The mean total BAI score significantly increased from 5.16 ± 6.59 points 1 month after the accident to 6.79 ± 8.41 points 7 months after the accident. (*p* < 0.05) (Table 2).

The proportion of the very severe impact of event group significantly decreased from 53 (7.5%) at 1 month after the accident to 26 (3.7%) at 7 months after the accident. The proportion of the severe impact of event group significantly decreased from 118 (16.6%) at 1 month after the accident to 69 (9.7%) at 7 months after the accident, and the proportion of the moderate impact of event group also decreased from 286 (40.2%) at 1 month after the accident to 204 (28.7%) at 7 months after the accident. On the other hand, the proportion of the normal impact of event group increased from 254 (35.7%) at 1 month after the accident to 412 (57.9%) 7 months after the accident (*p* < 0.05). The proportion of the normal anxiety group significantly decreased from 687 (96.6%) at 1 month after the accident to 670 (94.2%) at 7 months after the accident. The proportion of the severe anxiety group significantly increased from 3 (0.4%) at 1 month after the accident to 10 (1.4%) at 7 months after the accident, and the proportion of the extreme anxiety group also increased from 6 (0.8%) at 1 month after the accident to 16 (2.3%) at 7 months after the accident. (*p* = 0.037) (Table 3).

**Table 3:** Distribution of impact of event and anxiety levels after 1 month and 7 months

	1st survey (1 month)		2nd survey (7 month)		*p*-value^a^
	*n*	(%)	*n*	(%)	
Impact of Event Scale					
Normal	254	(35.7)	412	(57.9)	0.000
Moderate	286	(40.2)	204	(28.7)	
Severe	118	(16.6)	69	(9.7)	
Very severe	53	(7.5)	26	(3.7)	
Anxiety Level					
Normal	687	(96.6)	670	(94.2)	0.037
Anxiety	15	(2.1)	15	(2.1)	
Severe anxiety	3	(0.4)	10	(1.4)	
Extreme anxiety	6	(0.8)	16	(2.3)	
Total	711	(100.0)	711	(100.0)	

^a^ *p* value for McNemar’s test

The proportion of subjects with persistent symptoms at 7 months after the accident increased in accordance with age. Concerning gender, the proportion of female subjects with persistent symptoms was higher than that of males (23.2% and 14.6%, respectively). Regarding hydrogen fluoride exposure level, the impact of event levels and anxiety levels were higher as the proportion of subjects with persistent symptoms increased. (Table 4).

**Table 4:** Proportion of subjects with 7-month persistent symptom^a^

	Symptom(−)		Symptom(+)		*P*-value^b^
	*N*	(%)	*N*	(%)	
Ages					0.000
< 20	198	(98.0)	4	(2.0)	
20–29	98	(90.7)	10	(9.3)	
30–39	128	(83.1)	26	(16.9)	
40–49	105	(83.3)	21	(16.7)	
50–59	34	(57.6)	25	(42.4)	
60–69	11	(45.8)	13	(54.2)	
70–79	8	(25.8)	23	(74.2)	
≥ 80	2	(28.6)	5	(71.4)	
Gender					0.004
Male	379	(85.4)	65	(14.6)	
Female	205	(76.8)	62	(23.2)	
Exposure level					0.000
Extremely low	51	(98.1)	1	(1.9)	
Low	219	(92.4)	18	(7.6)	
Moderate	191	(83.4)	38	(16.6)	
High	123	(63.7)	70	(36.3)	
Impact of Event Scale					0.000
Normal	388	(94.2)	24	(5.8)	
Moderate	162	(79.4)	42	(20.6)	
Severe	29	(42.0)	40	(58.0)	
Very severe	5	(19.2)	21	(80.8)	
Anxiety Level					0.000
Normal	568	(84.8)	102	(15.2)	
Anxiety	8	(53.3)	7	(46.7)	
Severe anxiety	3	(30.0)	7	(70.0)	
Extreme anxiety	5	(31.3)	11	(68.8)	

^a^Pesistent symptom: one or more of the three symptoms (persistent irritation in the eyes, throat, respiratory system), these were identified as persistent physical symptoms

^b^ *p* value for chi-square test

Multiple logistic regression analysis was performed by using persistent physical symptoms as a dependent variable and age group, gender, hydrogen fluoride exposure level, and the impact of event and anxiety levels 7 months after the accident as independent variables. Independent variables that were significantly associated with the dependent variable were age group, gender, hydrogen fluoride exposure level, and the impact of event level. Concerning age group, the odds ratios were 3.994 (95% CI = 1.179–13.531), 7.199(95% CI = 2.242–23.120), 5.740(95% CI = 1.784–18.474), 15.263(95% CI = 4.471–52.098), 17.564(95% CI = 4.154–74.265), 39.326(95% CI = 9.674–159.870), and 35.498(95% CI = 3.549–355.050) for persistent symptoms in relation to subjects under the age of 20, and in their 20s, 30s, 40s, 50s, 60s, 70s, and 80s, respectively. The risk of persistent symptoms was relatively high among those in their 50s, 60s, 70s, and 80s. Concerning gender, the odds ratio was 2.001 (95% CI = 1.170–3.423) for female subjects in relation to male subjects. The risk of persistent symptoms was relatively higher in female. Regarding hydrogen fluoride exposure level, the odd ratios were 13.440(95% CI =1.118–161.547), 16.995(95% CI = 1.447–199.595), and 23.289(95% CI =2.005–270.526) for the low, moderate, and high exposure groups, respectively, in relation to persistent symptoms among the extremly low exposure group. The risk of persistent physical symptoms increased as hydrogen fluoride exposure level increased. Regarding the impact of event level, the odd ratios for persistent symptoms in relation to the normal group were 2.302(95% CI = 1.249–4.241), 5.268(95% CI = 2.397–11.575), and 27.834(95% CI = 5.747–134.811) for the moderate, severe, and very severe impact of event groups, respectively. The risk of persistent symptoms increased as the impact of event level increased (Table 5).

**Table 5:** Results of multiple logistic regression analysis for 7-month persistent symptoms

			95% CI^a^		
	Crude OR^b^	Adjusted OR^c^	Lower	Upper	*P*-value^d^
Ages					
< 20	1	1			
20–29	6.187	3.994	1.179	13.531	0.026
30–39	10.055	7.199	2.242	23.120	0.001
40–49	9.900	5.740	1.784	18.474	0.003
50–59	36.397	15.263	4.471	52.098	0.000
60–69	58.500	17.564	4.154	74.265	0.000
70–79	142.312	39.326	9.674	159.870	0.000
≥ 80	123.750	35.498	3.549	355.050	0.002
Gender					
Male	1	1			
Female	1.838	2.001	1.170	3.423	0.011
Exposure level					
Extremely low	1	1			
Low	4.700	13.440	1.118	161.547	0.041
Moderate	10.147	16.995	1.447	199.595	0.024
High	29.024	23.289	2.005	270.526	0.012
Impact of Event Scale					
Normal	1	1			
Moderate	4.134	2.572	1.439	4.596	0.001
Severe	21.352	5.811	2.725	12.391	0.000
Very severe	65.016	30.726	6.657	141.806	0.000
Anxiety level					
Normal	1	1			
Anxiety	4.762	1.166	0.322	4.216	0.815
Severe anxiety	12.699	1.619	0.278	9.409	0.592
Extreme anxiety	11.973	0.619	0.105	3.628	0.595

^a^ *CI* confidence interval

^b^Crude OR: Crude odds ratio

^c^Adjusted OR: Adjusted odds ratio for ages, gender, exposure level, Impact of event scale, Anxiety level

^d^ *p* value for multiple logistic regression

## Discussion

This study was conducted to evaluate the psychological effects of the hydrogen fluoride spill on residents and workers living near the accident area and to investigate changes in psychological status and the relationship between psychological effects and physical symptoms with the passage of time after the accident.

The mean IES-R-K score or the mean score of impact of event levels and the mean BAI score or the mean score of anxiety levels 1 month and 7 months after the accident significantly increased as hydrogen fluoride exposure level increased. The proportion of the participants within the very severe impact of the event group 7 months after the accident decreased, as did the mean score of the impact of event levels. The proportion of the respondents within the normal anxiety group 7 months after the accident decreased, and the mean score of the anxiety levels 7 months after the accident increased. The risk of persistent symptoms at 7 months after the accident was higher in females, and this risk significantly increased with increasing age, hydrogen fluoride exposure, and impact of event levels.

Among the previous studies concerning the psychological effects of a hydrogen fluoride spill on the surrounding community, there was one conducted after the 1987 Texas hydrogen fluoride spill accident. In that study, exposure level, physical symptoms, and psychological effects 1 and 2 years after the accident were investigated through surveys. Total psychological scores tended to increase as hydrogen fluoride exposure level increased. Physical symptoms after the accident were found to be the most closely related to hydrogen fluoride exposure level, but there were also significant effects on psychological status [13]. In the present study, psychological effects were assessed using the IES-R-K and the BAI, and the questionnaire items used in this study to assess psychological effects were different from those used in previous studies. However, the results of the present study showed that both the impact of event and anxiety levels increased as hydrogen fluoride exposure increased, and that the risk of persistent symptoms increased as hydrogen fluoride exposure and impact of event levels increased. These results were similar to the results of previous studies.

The disaster type differed between the present study and previous studies concerning the long-term course of PTSD after a disaster, but the finding that the prevalence of PTSD declined with the passage of time was common. The prevalence of PTSD among residents near the 9/11 terrorist attacks in New York declined from 17% after 2 months to 5.8% after 3 months following the attacks. The prevalence of PTSD among survivors of a plane crash in Alabama declined from 54% 1 month after the accident to 10—15% 1 year after the accident. The prevalence of PTSD among refugees in a village after the 2004 tsunami in Thailand declined from 12% 2 months after the tsunami to 7% 9 months after the tsunami [14–17]. The results of the present study showed that the proportion of subjects with very severe impact of event and the mean score of the impact of event levels decreased 7 months after the accident, which were similar to the results of previous studies.

Generalized anxiety disorder generally lasts for more than 6 months, and its main symptoms include excessive worry, anxiety, tension, and non-specific physical symptoms. A previous study reported that only 20% of patients with generalized anxiety disorder can be completely treated, and the disease has a duration of 5 to 10 years. Patients with generalized anxiety disorder excessively use primary medical institutions, rather than psychiatric care, thus resulting in a personal financial burden. In addition, if accompanied by mental illnesses such as depression, specific phobias, and PTSD, patients with generalized anxiety disorder may become a social burden due to their loss of social and occupational functioning [18]. As shown in Table 2, the mean score of anxiety levels 7 months after the hydrogen fluoride exposure accident increased in all groups, confirming that anxiety level did not resolve but persisted even with the passage of time after the accident The reason for this is that the anxiety disorder generally lasts more than 6 months and lasts for more than years. In this study, it seems that the choice of 7 months past the spillage was not enough time for the respondents’ symptoms to resolve. In addition, psychological counseling and intervention were rarely carried out since attention was only given to the physical effects of the spillage on the participants after the accident. If there had been a proper psychological intervention, there would have been a reduction in the levels of anxiety even if complete recovery could not be achieved. When making comparisons based on the hydrogen fluoride exposure levels, the results showed that the mean score of anxiety levels was 3.64 points higher at 7 months after the accident than it was at 1 month after the accident among the high exposure group. Furthermore, this increment in the mean score was greater in the high exposure group than that in the groups with extremely low, low, and moderate exposures. These findings suggest that although anxiety levels are high during the early stages of an accident, it may even be greater afterward. Therefore, psychological interventions are considered necessary for high risk groups during the early stages of an accident so as to prevent chronicity.

In meta-analysis studies on psychological interventions for the victims of disasters and accidents, several intervention methods were found to help the rapid recovery from psychological problems. Specifically, crisis intervention, in which the victims of a disaster or accident discuss their thoughts and feelings with a psychologist and are counseled in a supportive manner within a few weeks after an accident helps them recover quickly, as does providing the victims with accurate accident-related information following the accident. Cognitive therapy performed several months after an accident was found to lower the incidence of PTSD [19]. In order to prevent long-term psychological effects of hydrogen fluoride exposure on the community, it is considered necessary to evaluate the psychological effects at the early stages of an accident and implement active interventions for high-risk groups as a result of evaluations. In addition, it will also be necessary for expert groups and related organizations to provide accurate information concerning accident substances and situations so as to relieve anxiety.

Recent studies have reported that psychological shock and PTSD are associated with an increased risk of cardiovascular disease, gastrointestinal disease, chronic fatigue, and musculoskeletal diseases, rather than non-specific symptoms. Those studies explained how psychological shock acts on the hypothalamic-pituitary-adrenal (HPA) axis or the sympathetic-adrenal-medullary (SAM) stress axis, causing psychological effects. In addition, changes in the HPA and SAM axes in the PTSD patients affected the neuroendocrine function, resulting in elevated leukocyte and total T-cell counts and reduced natural killer cell cytotoxicity, leading to inflammation and immunosuppression [20]. In a study of firefighters who were exposed to the 1983 bushfire disaster in Australia, those with PTSD were found to be more likely to complain of cardiovascular, respiratory, musculoskeletal, and neurological symptoms than those who did not experience PTSD [21]. The present study also found that the risk of persistent physical symptoms increased with increasing impact of event level, which was similar to the results of previous studies. PTSD-induced inflammation and immunosuppression may cause eye, nose, and throat disorders, which may have affected the persistence of physical symptoms. Therefore, it is thought that the implementation of active psychological interventions for individuals with very severe impact of event levels at the early stages of an accident would be helpful in resolving physical symptoms afterwards.

The present study has limitations. First, although individual sample measurements or atmospheric concentration measurements had to be performed to assess accurate exposure levels at the time of the accident, they could not be performed due to the sudden nature of the accident. Exposure levels were indirectly estimated through a questionnaire; thus, the estimated levels may be different from the actual exposure levels. Second, in order to assess the psychological effects, surveys including the IES-R-K and the BAI were conducted in lieu of a diagnosis. Although a diagnosis of PTSD or anxiety disorder is made through structured interviews with a psychiatrist, such a diagnosis was replaced by surveys in the present study. Therefore, the prevalence of actual diseases is not accurately reflected. Third, psychological effects can evidence themselves over several years. In this regard, another limitation is that although the psychological effects were analyzed two times at 1 month and 7 months after the accident, long-term effects were not assessed in the present study. Fourth, there were 648 missing subjects in the second survey, with a possibility of selection bias. The psychological scores of the first survey were compared between the group of 711 subjects who participated in both the first and second surveys and the group of 648 subjects who participated only in the first survey. The mean IES-R-K scores were 33.33 ± 14.64 and 36.54 ± 16.85, respectively, which were lower in the 711 subjects group. The mean BAI scores were 5.16 ± 6.59 and 5.55 ± 7.28, respectively but not significantly different. Although we compared the 711 paired subjects, it was thought that if there were more subjects in the second survey, the result of the impact of event level change over time would have been different.

Unlike other studies that focused on the physical effects after the Gumi hydrogen fluoride spill in 2012, the present study is significant in that it yielded meaningful results concerning the psychological effects of hydrogen fluoride exposure and the changes with the passage of time after the accident.

It is necessary for concerned authorities to evaluate the psychological effects of a disaster on community from the outset of the disaster. After the evaluation, the implementation of active interventions for the high-risk group will be helpful in reducing the physical and psychological damage. The present study revealed that psychological effects were greater among subjects with higher hydrogen fluoride exposure levels. It was also found that the female subjects, elderly subjects, and subjects with a high impact of event level were more likely to have persistent physical symptoms. Therefore, more active management is considered to be necessary for these groups after an accident. Furthermore, it was found that the impact of event level decreased after the accident, whereas anxiety level tended to increase with the passage of time after the accident. It is considered necessary for expert groups and related authorities to provide accurate information to the community.

## Conclusion

The present study found that the impact of event and anxiety levels increased with increasing hydrogen fluoride exposure levels, as was the case with the passage of time after the accident. The impact of event level was found to decrease with the passage of time after the accident, whereas anxiety level was found to increase. The risk of persistent physical symptoms was higher in females, and it increased with increasing age, hydrogen fluoride exposure level, and impact of event levels.

From the results of the present study, we could find that it is very important for experts and related authorities to provide the community with accurate information in the early stages following a chemical accident so as to relieve the community’s anxiety. In addition, it is thought that assessing the psychological effects of a chemical accident on community and implementing active interventions for high-risk groups in the early stages of such a chemical accident will contribute to reducing physical and psychological damage.

## Abbreviations

BAI: Beck anxiety inventory
HPA: Hypothalamic-pituitary-adrenal
IES-R-K: Impact of event scale – revised korean version
PTSD: Post traumatic stress disorder
SAM: Sympathetic-adrenal-medullary
